# Toxocariasis Diagnosed in International Travelers at the Institute of Tropical Medicine, Antwerp, Belgium, from 2000 to 2013

**DOI:** 10.1371/journal.pntd.0003559

**Published:** 2015-03-06

**Authors:** Steven Van Den Broucke, Kirezi Kanobana, Katja Polman, Patrick Soentjens, Marc Vekemans, Caroline Theunissen, Erika Vlieghe, Marjan Van Esbroeck, Jan Jacobs, Erwin Van Den Enden, Jef Van Den Ende, Alfons Van Gompel, Jan Clerinx, Emmanuel Bottieau

**Affiliations:** 1 Department of Clinical Sciences, Institute of Tropical Medicine, Antwerp, Belgium; 2 Department of Biomedical Sciences, Institute of Tropical Medicine, Antwerp, Belgium; University of Melbourne, AUSTRALIA

## Abstract

Although infection with *Toxocara canis* or *T*. *catis* (commonly referred as toxocariasis) appears to be highly prevalent in (sub)tropical countries, information on its frequency and presentation in returning travelers and migrants is scarce. In this study, we reviewed all cases of asymptomatic and symptomatic toxocariasis diagnosed during post-travel consultations at the reference travel clinic of the Institute of Tropical Medicine, Antwerp, Belgium. Toxocariasis was considered as highly probable if serum *Toxocara*-antibodies were detected in combination with symptoms of visceral larva migrans if present, elevated eosinophil count in blood or other relevant fluid and reasonable exclusion of alternative diagnosis, or definitive in case of documented seroconversion. From 2000 to 2013, 190 travelers showed *Toxocara*-antibodies, of a total of 3436 for whom the test was requested (5.5%). Toxocariasis was diagnosed in 28 cases (23 symptomatic and 5 asymptomatic) including 21 highly probable and 7 definitive. All but one patients were adults. Africa and Asia were the place of acquisition for 10 and 9 cases, respectively. Twelve patients (43%) were short-term travelers (< 1 month). Symptoms, when present, developed during travel or within 8 weeks maximum after return, and included abdominal complaints (11/23 symptomatic patients, 48%), respiratory symptoms and skin abnormalities (10 each, 43%) and fever (9, 39%), often in combination. Two patients were diagnosed with transverse myelitis. At presentation, the median blood eosinophil count was 1720/μL [range: 510–14160] in the 21 symptomatic cases without neurological complication and 2080/μL [range: 1100–2970] in the 5 asymptomatic individuals. All patients recovered either spontaneously or with an anti-helminthic treatment (mostly a 5-day course of albendazole), except both neurological cases who kept sequelae despite repeated treatments and prolonged corticotherapy. Toxocariasis has to be considered in travelers returning from a (sub)tropical stay with varying clinical manifestations or eosinophilia. Prognosis appears favorable with adequate treatment except in case of neurological involvement.

## Author Summary

Toxocariasis is a zoonosis of worldwide distribution caused by dog (*Toxocara canis*) or cat (*T*. *catis*) roundworm that can be fully asymptomatic or may cause significant disease such as a the systemic syndrome called visceral larva migrans as well as neurological or eye manifestations. Toxocariasis prevails in tropical areas, but information about this disease in travelers and migrants is scarce. In this study, we describe in detail a case series of 28 international travelers, mostly adults, diagnosed with toxocariasis from 2000 to 2013 at the reference travel clinic of the Institute of Tropical Medicine of Antwerp, Belgium. We found this infection in all types of travelers returning from any part of the world. Clinical symptoms, when present, varied widely and an increase of the blood eosinophil count was almost always present. Morbidity was substantial and 2 patients had severe neurological complications. Diagnosis was difficult in travelers because the illness often resembled other tropical infections. Recovery was, however, complete, either spontaneously or with anti-parasitic drugs, except in both cases with neurological involvement. Toxocariasis is one of the numerous parasitic infections to consider in travelers returning from the tropics with any type of symptoms or with an increased blood eosinophil count.

## Introduction

Toxocariasis, caused by intestinal roundworm of dogs (*Toxocara canis*) or cats (*Toxocara catis*), is a zoonotic infection with a worldwide distribution [1,2]. Humans can get infected by ingestion of embryonated eggs present on the soil, plants or soil-dwelling invertebrates contaminated by dog or cat feces, and less frequently by ingestion of encapsulated larvae from undercooked paratenic hosts such as chickens, cattle and sheep. Infection is followed by the migration of third-stage larvae through the tissues which is usually asymptomatic but may be associated with a variety of non-specific clinical features [2,3]. Presentation may be acute or sub-acute, with systemic, abdominal or respiratory manifestations, classically described as the syndrome of visceral larva migrans (VLM) sometimes associated with dermatological symptoms as well. Occasionally, the course is complicated by the involvement of the central nervous system (neurological toxocariasis) or the eyes (ocular toxocariasis). Like in other helminthic infections with larval migration, blood eosinophilia is common and/or numbers of eosinophils may be increased in other relevant tissues or fluids; other laboratory abnormalities such as elevated inflammatory parameters or liver enzymes disturbances are often mild and inconstant. Of note, a distinct clinical presentation called “covert” (in children) or “common” (in adults) toxocariasis has been described more recently, with more subtle and chronic symptoms such as cough, abdominal pain or pruritus, associated with mild eosinophilia and *Toxocara spp*. seropositivity [4–6]. It is however assumed that most cases of human toxocariasis go unrecognized.

A definitive diagnosis of human toxocariasis can only be made by histological examination of infected tissue, demonstrating *Toxocara spp*. larvae within eosinophilic granulomatous lesions. Since sampling of appropriate tissue is rarely justified on clinical grounds, diagnosis of human toxocariasis relies in the daily practice on a constellation of suggestive symptoms (if present), combined with laboratory abnormalities (blood eosinophilia) and the detection of circulating immunoglobulin G (IgG) antibodies to *Toxocara* excretory–secretory (TES) antigens [7]. In Western countries with low prevalence of helminthic infections, the association of clinical symptoms (although frequently poorly specific) with eosinophilia usually triggers clinicians aware of the disease to perform the specific serological investigations [1]. In the tropics however, where polyparasitism is highly prevalent, the low specificity of eosinophilia, the lack of diagnostic facilities and the cross-reactivity with other helminthic infections often preclude the etiologic diagnosis [2].

While larvae may remain viable for years in the tissues, human toxocariasis is usually a self-limiting disease, although its course can sometimes be invalidating and prolonged (up to several weeks). Treatment firstly aims at controlling the inflammatory reaction when needed, and despite the limited knowledge about its clinical benefit, anti-helminthic therapy is often associated. In such cases, preference usually goes to albendazole for its parasitological efficacy and good clinical tolerance [8].

The global burden of human toxocariasis is poorly quantified [9]. Serological surveys demonstrate that the infection is more frequent in tropical settings and in rural areas, with population-based seroprevalence ranging from 2.5% in urban Europe up to 85% in rural tropics [9–14]. Visceral larva migrans has been reported in individuals born in tropical countries having migrated to Europe [15]. However, although many susceptible European travelers are assumed to be at increased risk of exposure to *Toxocara* spp. during a stay in highly endemic developing countries, frequency and presentation of toxocariasis are largely unknown in this population. A recent large multicenter GeoSentinel study has reported 16 cases diagnosed as visceral larva migrans among 42,173 travelers evaluated from 2007 to 2011 (38 cases per 100,000 travelers) but there was no standard diagnostic approach in the 53 participating travel clinics and no clinical description [16]. In the present study, we aimed to assess the frequency, presentation and outcome of toxocariasis diagnosed among travelers and migrants presenting at the travel clinic of the Institute of Tropical Medicine of Antwerp, Belgium.

## Materials and Methods

### Study Setting and Population

For this retrospective study, a query was first undertaken in the database of the Central Laboratory of Clinical Biology of the Institute of Tropical Medicine, Antwerp (ITMA), to retrieve all results of *Toxocara* serology requested in patients having attended the travel clinic of the ITMA from 2000 to 2013. The ITMA is the national reference center for tropical medicine in Belgium, with on average about 6500 consultations a year for post-travel care. The medical records of all travelers and migrants found with a positive *Toxocara* serology during this period were then reviewed. Relevant clinical and laboratory data were extracted, de-identified and entered in a Microsoft Access 2010 database. Variables included: demographic data, month and year of first *Toxocara* positive serology, most recent travel destination, time of symptom onset after travel, duration of symptoms before consultation, clinical presentation, result of the chest X-rays if requested, absolute blood eosinophil count (and percentage of white blood cell count), *Toxocara* antibody optical density result, results of parasitological and serological tests prescribed by the physician targeting other helminths according to epidemiological relevance (*Ascaris spp*., *Echinococcus granulosus*, *Fasciola spp*., *Filaria spp*., *Schistosoma spp*., *Taenia solium*, *Strongyloides stercoralis*, *Trichinella spp*. and *Anisakis simplex*), administered treatment(s), clinical and laboratory evolution and outcome.

**Fig 1:**
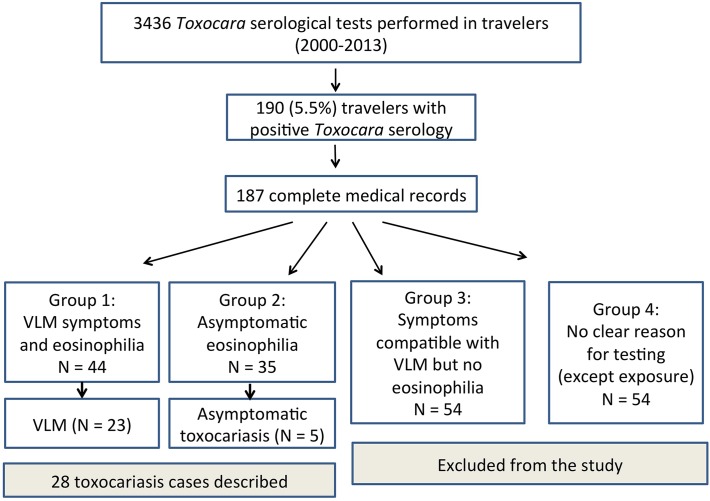
Flow chart and distribution of study participants (VLM: visceral larva migrans).

Next, we categorized all reviewed *Toxocara*-seropositive cases in four groups according to the clinical reasons for requesting *Toxocara* serology: 1) presence of symptoms compatible with VLM combined with eosinophilia; 2) asymptomatic eosinophilia; 3) symptoms compatible with VLM without eosinophilia; and 4) possible exposure/no clear reason (Fig. 1).

### Definition of Human Toxocariasis

For this clinical study, strict case definitions of symptomatic (*Toxocara*-associated visceral larva migrans) and asymptomatic toxocariasis were used. Diagnosis of *Toxocara*-associated VLM was considered as highly probable when the following criteria were all fulfilled: (1) positive *Toxocara* serology (entry criteria) AND (2) presence of any systemic symptom compatible with toxocariasis (including fever, respiratory signs such as wheezing, dry cough, dyspnea or an infiltrate on the chest X-ray, abdominal symptoms as abdominal pain, vomiting, diarrhea or hepatomegaly, neurological signs such as focal deficit or encephalopathy, and ocular signs such visual disturbances), with or without dermatological symptoms such as pruritus, urticarial rash or angio-edema AND (3) blood eosinophilia (defined as an absolute blood eosinophil count above 500/μL, or > 7% of the white blood cell count at first presentation [17,18]), or presence of eosinophils in another relevant fluid/tissue) AND (4) reasonable exclusion of alternative diagnosis. Diagnosis of VLM was considered definitive when all 4 criteria were present together with unequivocal *Toxocara* seroconversion documented on paired serum samples. Diagnosis of asymptomatic toxocariasis relied on the presence of blood eosinophilia at presentation in asymptomatic *Toxocara*-seropositive individuals and no evidence of other infection likely to explain the eosinophilia.

To comply with the case definition, we therefore did not further analyze groups 3 and 4 (since there was no eosinophilia), and then carefully assessed for all cases of groups 1 and 2 whether an alternative diagnosis or a co-infection were possible. In accordance with the case definitions, we finally excluded from this study all patients with parasitological or serological evidence of another infection or clinically suspect of alternative diagnosis (such as allergy, scabies,…).

### Serological Testing

From 2000 to 2009, the serological diagnosis of toxocariasis was performed with a commercial anti-TES IgG enzyme-linked immunosorbent assay (ELISA) (*Toxocara canis*, Bordier Affinity Products SA, Crissier, Switzerland) according to the instructions of the manufacturer. The assay’s threshold for positivity was set as “weak positive” (from 2000 to 2009) and at 1.0 (measured by optical density, since 2010 onwards) for use in clinical settings [13]. In this context, the assay has been reported to provide a sensitivity and a specificity of 91% and 86% respectively, the latter being evaluated in a population of patients with protozoan and helminthic infections [19].

### Case Management

When toxocariasis is strongly suspected or confirmed, the standard treatment regimen at ITMA is albendazole 2 x 400 mg (in adults) for 5 days, according to international guidelines. Corticosteroids may be added at the physician’s discretion according to severity of symptoms. Diethylcarbamazine (DEC) is restricted as second line therapy for refractory cases [20,21].

### Statistical Analysis

Analysis was performed with the SPSS program version 20.0 (SPSS Inc., Chicago, IL, USA). Differences were compared using Student’s *t*-test or Mann-Whitney *U*-test, when appropriate, for continuous outcome and chi-square and Fisher’s exact tests for categorical outcomes. *P* values of less than 0.05 were considered to indicate statistical significance.

### Ethics Statement

This was a retrospective analysis of data collected during routine clinical care over a 13-year period. Ethical clearance was obtained from the institutional review board at ITMA. Laboratory queries were obtained in an anonymous way. Clinical data were then retrieved through an encoded link and de-identified for analysis according to the Belgian legislation. No written informed consent was obtained from individual participants because an opt-out strategy is in place at ITMA covering surveillance use of clinical and laboratory data.

## Results

### Diagnosis of Toxocariasis in Travelers

From January 2000 to August 2013, 3436 *Toxocara* serological tests were ordered for diagnostic purpose in post-travel care by the ITMA physicians. Of these tests, 190 (5.5%) had positive anti-TES IgG (Fig. 1). Of 187 patients with complete clinical data, 44 had VLM symptoms and eosinophilia (group 1), 35 were found with asymptomatic eosinophilia (group 2) and 54 had symptoms compatible with VLM but no eosinophilia (group 3, excluded from the study); for the remaining 54 individuals with no symptoms and no eosinophilia, the reason for *Toxocara* testing was considered as unclear (group 4, also excluded from further analysis).

In the groups 1 and 2, after exclusion of the cases with alternative diagnosis or possible co-infection, a diagnosis of human toxocariasis was retained in 28 patients, including *Toxocara*-associated VLM in 23 and asymptomatic toxocariasis in 5 (Fig. 1). Diagnosis of VLM was considered as definitive in 7 cases for whom seroconversion was observed. Histological examination was not performed in any case. Of note alternative diagnoses in the excluded cases of groups 1 and 2 (n = 51) were mainly strongyloidiasis (n = 15), allergic reaction (n = 7), filarial infection (n = 7), *Ancylostoma*/*Ascaris* infection (n = 7) and schistosomiasis (n = 6). Suspicion of co-infection was also frequent.

### Epidemiological, Clinical and Laboratory Features of Toxocariasis Cases

The clinical presentation of the 28 cases is detailed in Table 1. Cases were evenly distributed throughout the study period with no cluster phenomenon. All patients were adult travelers born or residing in Europe, except one Ethiopian child evaluated after adoption and one Lebanese adult living in the Democratic Republic of the Congo. Mean age was 46 years (range: 4–68 years) with a male/female ratio of 0.87. Regions of most recent travels (and presumed exposure) were North and sub-Saharan Africa in 10 patients, Southern and Southeast Asia in 9 and Southern Europe (including Turkey) in 6; for 3 patients (numbers 18, 19 and 26), the continent of acquisition could not be traced with certainty because of multiple travel destinations within a short timeframe. Duration of travel was less than 1 month in 12 (43%) patients and more than 3 months in 10 (36%).

**Table 1:** Epidemiological, clinical, laboratory and outcome features of patients diagnosed with toxocariasis (n = 28) at the Institute of Tropical Medicine, Antwerp, Belgium from 2000 to 2013.

Case n°	Date of diagnosis	Age, year/ gender	Origin	Travel destination (duration)	Symptoms at presentation	Symptom duration	Interval travel-symptom onset or screening	Absolute eosinophil count at first documented assessment (% of WBC)	Toxocara serology (+ titer since 2010)	Initial treatment, evolution and outcome (interval for follow-up)	Comments
1	April 2000	45/M	Belgium	Cambodia (10 months)	Abdominal pain, cough, wheezing	1 week	During stay	3560 (25%) (in Cambodia)	Negative (in Cambodia)	Praziquantel 2 days, with no clinical improvement; albendazole thereafter for 3 weeks Clinical cure and normalization of eosinophil count (assessed in Belgium)	Documented seroconversion (week 5 post-symptom onset in Belgium)
2	May 2000	23/F	Belgium	Laos, Cambodia (2 months)	Fever, abdominal pain, diarrhea, urticarial rash	10 days	3 weeks after return	14160 (57%)	Positive	Albendazole 5 days, with clinical cure and normalization of eosinophil count (in 2002)	Spontaneous clinical improvement before therapy
3	Novem-ber 2000	23/F	Belgium	Indonesia (2 years)	none	-	12 days after return	2080 (23%)	Positive	Ivermectine and praziquantel, with normalization of eosinophil count (assessed at month 6 post-treatment)	No albendazole because abroad again when serological results became available
4	June 2002	35/F	Belgium	Southeast Asia (1 year)	Fever, vomiting, abdominal pain, cough, bronchitis	8 weeks	End of stay	730 (9%)	Positive	No treatment first, but no clinical improvement after one month; albendazole 5 days thereafter Clinical cure and normalization of eosinophil count (assessed at week 8 post-treatment)	
5	Augustus 2002	51/F	Belgium	Spain (1 week)	Cough and thoracic pain ; pruritus; no improvement with antibiotics	3 weeks	End of stay	2320 (24%)	Positive	Albendazole 5 days, with clinical cure and normalization of eosinophil count (assessed at week 4 post-treatment)	Spontaneous decrease of eosinophil count before therapy (till 920, 10%); Clear exposure to young dogs during travel
6	January 2003	52/M	Belgium	Multiple shorts stay in African countries	Dry cough, fever	3 weeks	5 weeks after return	1070 (16%)	Positive	Albendazole 5 days, with clinical cure and normalization of eosinophil count (assessed at week 3 post-treatment)	Spontaneous decrease of eosinophil count before therapy (till 590, 9%)
7	February 2003	37/M	Belgium	Nepal (8 months)	Pruritus and urticarial rash	6 weeks	End of stay	510 (9%)	Positive	Ivermectin 1 day, with no improvement; albendazole 5 days when results available Clinical cure thereafter	Clinical follow-up by phone No laboratory control
8	March 2003	42/M	Belgium	Romania (1 year)	Diarrhea, abdominal pain, night sweats, dyspnea	12 weeks	During stay	950 (11%)	Positive	Albendazole 5 days with clinical cure and normalization of eosinophil count (assessed at month 6 post-treatment)	
9	April 2004	49/M	Belgium	Laos (1 year)	Fever, abdominal pain, diarrhea	5 days	1 week after return	7600 (55%)	Negative	Albendazole 5 days, with clinical cure and normalization of eosinophil count (assessed at week 4 post-treatment)	Documented seroconversion (week 4 post-treatment)
10	January 2007	52/F	Belgium	South Africa (2 weeks)	Localized edema, urticarial rash, abdominal pain	10 days	4 weeks after return	2340 (26%)	Negative	Albendazole 5 days (week 2 after first contact), with clinical cure and normalization of eosinophil count (assessed at week 6 post-treatment)	Spontaneous decrease eosinophil count before therapy (till 990, 13%) Documented seroconversion (week 6 post-treatment)
11	July 2007	46/F	Belgium	Slovenia (2 weeks)	Paresthesia, followed by anesthesia in both legs and genital region; transient loss of strength	10 weeks	? (precise dates of travel not found)	490 (5%)	Positive	Albendazole 5 days + steroids; DEC administered after 1 week Slow clinical improvement Resolution of MRI lesions	*Toxocara* ELISA positive in CSF CSF examination: 33 WBC with 12% eosinophils Transverse myelitis at MRI
12	December 2007	53/F	Belgium	China (10 days)	no	-	1 month after return	1410 (18%)	Positive	Ivermectin 1 day, with no eosinophil decline 2 weeks later: 1300 (19%) Albendazole 5 days thereafter, with normalization of eosinophil count (assessed at week 8 post-treatment)	
13	February 2009	49/M	Belgium	Kenya (1 month) + other neighboring countries	Urticarial rash + intermittent edema face and hands, sweats	8 weeks	End of stay	670 (10%)	Positive	Albendazole 5 days, with clinical cure and normalization of eosinophil count (week 5 post-treatment)	Laboratory control in another institution
14	February2009	49/M	Belgium	Philippines (2 weeks)	Fever, cough dyspnea, wheezing	12 days	Day of return	1640 (13%)	Negative	Mebendazole 3 days + ivermectine 1 day, with clinical cure and normalization of eosinophil count (assessed at week 2 post-treatment)	Documented seroconversion (2 week post-treatment) Chest X-rays: two infiltrates
15	April 2009	52/F	Belgium	Turkey (3 weeks)	Abdominal pain and intermittent diarrhea	16 weeks	1 week after return	1720 (19%)	Positive	Albendazole 5 days	Lost to follow-up
16	December 2009	49/M	Belgium	Several short trips to Central Africa	Urinary and fecal incontinency; sexual dysfunction	28 weeks	6 weeks after one of the trips	430 (4%)	Positive	Albendazole + steroids ; later DEC, with slight clinical improvement and resolution of MRI lesions	*Toxocara* ELISA positive in CSF CSF examination: 40 WBC with 18% eosinophils Transverse myelitis at MRI (thoracic vertebra 5^th^ till 11^th^)
17	November 2010	52/F	Lebanon (lives in Belgium)	Angola (3 months)	Urticarial rash, fatigue, sweats	4 weeks	4 weeks after return	2950 (32%)	Positive (3.7)	Albendazole 5 days, with clinical cure and normalization of eosinophil count (assessed at week 14 post-treatment)	Spontaneous decrease before therapy (till 860, 11%)
18	April 2011	40/F	Belgium	Bolivie or Nepal (1 month)	Fever, cough, urticarial rash	4 weeks	4 weeks after return	1810 (24%)	Positive (3,8)	Albendazole 3 days only, with decrease of eosinophil count after one month (710, 12%) but re-increase in September to initial levels Start DEC and normalization of eosinophil count (assessed at week 6 post-treatment)	Albendazole badly tolerated (vomiting, protracted anosmia); stopped after 3 days Chest X-rays: infiltrates
19	May 2011	35/F	Netherlands	Stewardess (Netherlands Antilla (4 days, Laos (3 days, Ghana 3 days)	Abdominal pain, diarrhea, vomiting, thoracic pain and dyspnea; no improvement with antibiotics	3 weeks	1 week after trip to Antilla	10130 (54%)	Positive (1.6)	Ivermectin 1 day; albendazole 5 days when results available, with clinical cure and normalization of eosinophil count (assessed at week 3 post-treatment)	Chest X-rays: infiltrates
20	June 2011	68/F	Belgium	Egypt (3 weeks)	Cough and wheezing	8 weeks	End of stay	540 (7%)	Positive (3.5)	Albendazole 5 days, with clinical cure and normalization of eosinophil count (assessed at week 2 post-treatment)	CT Scan thorax : infiltrates Chest X-rays: normal
21	November 2011	57/M	Lebanon	Democratic Republic of the Congo (residence)	no		Just after return	2590 (23%)	Positive (2.3)	Albendazole 5 days, with normalization of eosinophil count documented (assessed at week 8 post-treatment)	Laboratory control in another clinic
22	April 2012	65/F	Belgium	India (10 weeks)	Cough, fever, arthralgia, diarrhea, vomiting, itching;	4 weeks	End of stay	5850 (42%)	Negative (in Italy)	Albendazole 5 days, 2 weeks later in Belgium, with clinical cure and normalization of eosinophil count (assessed at week 3 post-treatment)	Admission 6 days in Italy first where steroids were required Chest X-rays (Italy): infiltrates Documented seroconversion (*Toxocara* titer 1.6; 4 weeks after symptom onset)
23	June 2012	4/F	Ethiopia	Ethiopia (residence)	no		At arrival (adoption)	1100 (11%)	Positive (1.6)	Albendazole 5 days, with normalization of eosinophil count (assessed at week 7 post-treatment)	
24	June 2012	64/F	Belgium	Turkey (4 months)	no		Return 1 month ago	2970 (26%)	Positive (3.1)	Albendazole 5 days, with normalization of eosinophil count (assessed at week 9 post-treatment)	Spontaneous decrease (till 1190, 11%) before therapy
25	June 2012	31/M	Belgium	Pilote ; Central/West Africa	Abdominal pain and diarrhea	10 days	Upon return	1260 (15%)	Positive (1.1)	Albendazole 5 days, with clinical cure and normalization of eosinophil count (assessed at week 4 post-treatment)	*Shigella* co-infection
26	September 2012	52/M	Belgium	Madagascar (14 months)	Abdominal pain, urticarial, quincke edema	4 weeks	During stay	780 (8%)	Positive (1.3)	No treatment Spontaneous cure assessed at week 4	
27	May 2013	66/M	Belgium	Burkina Faso (2 weeks) or France (1 month) ?	Fever, urticarial, lymphadenopathies	5 days	8 weeks (Burkina); 2 weeks (France)	3110 (33%)	Negative (0.7)	No treatment Spontaneous cure assessed at week 3 (280, 5%)	Documented seroconversion at week 3 post-symptom onset (titer 1.1)
28	July 2013	53/M	Belgium	Italy	Fever, night sweats, arthralgia,	3 weeks	10 days	1350 (16%)	Positive (1.6)	No treatment Spontaneous cure assessed at week 4	

Note: rows 3, 12, 21, 23, and 24 represent asymptomatic cases

M denotes male; F female; WBC white blood cell; CSF cerebrospinal fluid; MRI magnetic resonance imaging; DEC diethylcarbamazine

For the 23 cases presenting with VLM, symptoms started during the stay abroad in 11 (48%) or developed within 3 weeks in average (range: 0–8 weeks) after return. For one patient (number 11), the dates of recent travel were not clearly reported. Clinical manifestations included abdominal complaints in 11 (48%) patients, respiratory symptoms and skin abnormalities in 10 (43%) each and fever in 9 (39%); in most cases, symptoms were combined or developed sequentially. Radiological pneumonia was found in 5 patients and one of them (number 22) had to be admitted elsewhere because of the severity of respiratory symptoms. Two patients (numbers 11 and 16) presented with progressive neurological features of transverse myelitis but no other symptoms. In both cases, the diagnosis was made by the demonstration of an increased eosinophil count and of anti-TES IgG in the cerebrospinal fluid, in the absence of another etiology (Table 1).

For the 21 patients without neurological complications, median duration of symptoms before first documented medical evaluation at ITMA or elsewhere was 3 weeks (range 5 days- 4 months), while both patients with neurological toxocariasis were diagnosed several months (2,5 and 7 respectively) after symptom onset.

At first presentation, the median blood eosinophil count was 1720/μL (range: 510–14160) in the 21 VLM cases without neurological complication and 2080/μL (range: 1100–2970) in the 5 asymptomatic cases. Of note, the median eosinophil count was significantly lower in the VLM patients presenting more than 4 weeks after symptom onset than in those consulting earlier (700/μL versus 2340/μL, p<0.001). The blood eosinophil count was within the reference ranges for both patients with neurological toxocariasis.

### Treatment, Evolution and Outcome

Of the 5 patients with asymptomatic toxocariasis, 3 received the 5-day course of albendazole upon diagnosis, and the eosinophil count normalized uneventfully; the other 2 patients were empirically treated before the complete results were available: one with ivermectin (who required albendazole secondarily because eosinophilia persisted) and the second with ivermectin and praziquantel (with resolution of the eosinophilia).

Of the 23 symptomatic cases, 15 received a standard course of albendazole within one or two weeks after presentation (when laboratory results were available): 11 clinically recovered and eosinophil counts normalized (of note 4 had already clinically improved before the start of therapy); one was lost to follow-up; another had to switch to DEC because of immediate anosmia attributed to albendazole; both neurological cases were given steroids concomitantly with albendazole, but since the improvement was slow DEC was also administered (with resolution of the radiological abnormalities and moderate clinical recovery). Four patients were initially treated empirically with praziquantel (n = 1), or ivermectine (n = 3) but 3 of them required albendazole secondarily in the absence of substantial clinical/laboratory improvement. Finally the remaining 4 patients preferred at first not to be treated; 3 of them recovered spontaneously but the 4^th^ patient needed a course of albendazole a few weeks later and persisting symptoms eventually subsided. Of note only one non-neurological patient (number 22) required corticosteroids during hospitalization to control the severe respiratory symptoms.

## Discussion

Although toxocariasis is highly prevalent in most tropical areas, this condition has been hardly studied in returning travelers and migrants. We describe here a case series of 28 patients diagnosed with toxocariasis acquired from all over the world. Clinical presentation was extremely varied and resembled that of many other endemic helminth infections. Morbidity was important and complications sometimes serious.

This study has many limitations. It was indeed a retrospective single-center study conducted in a reference travel clinic, meaning that collection of data was not systematic and that findings may not be generalizable to all clinical settings. For instance, some cases with longer incubation period may have been directly attended in the primary care setting given the fact that the link between symptoms and travel had become less obvious. Also our observations are not transposable as such to the features observed in autochthonous toxocariasis sporadically seen in Belgium or elsewhere, mainly in children [22]. Other limitations could be related to the restrictive case definition that may have missed several true cases without eosinophilia (as sometimes observed in milder cases of “covert/common” toxocariasis) or with negative *Toxocara* serology (during the serological window period or because sensitivity does not reach 100%, in particular in low burden infection such as ocular toxocariasis [2]). In the same line, some true toxocariasis cases may have been disregarded just because serological tests against other helminths were also positive, either by cross-reactivity or as part of infection with multiple parasites. Conversely, false positive result was also possible if anti-*Toxocara* seropositivity was just reflecting remote exposure or cross-reaction while another helminthic infection was missed during the workup. The commercial test we used is widely considered as an adequate screening tool for clinical practice but indeed detects anti-TES IgG that may persist for years; IgM or IgE-based serological tests that could better discriminate recent infection are not routinely available. On the other side, for European travelers with little previous exposure to parasites, cross-reaction is probably not a major issue, since test threshold has been set at value providing good specificity. In addition, the consistent clinical and laboratory diagnostic approach by a stable group of expert physicians throughout the study probably reflected the best accuracy that can be obtained in routine care. Finally, in this series, infection was most likely acquired abroad since symptoms developed during or shortly after travel, but infection in Belgium before travel or after return cannot be fully excluded.

The observation of toxocariasis in travelers is not surprising, although poorly studied so far. In a 10-year retrospective study in Spain, *Toxocara* antibodies were detected in 31 (4.9%) of 634 Latin American migrants and VLM was diagnosed in 4 of them [15], but this was in a particular segment of the travel population. With 28 highly probable/definitive toxocariasis cases diagnosed in about 85,000 travelers during the 13-year study period (33/100,000 travelers), our findings are in line with the recent multicenter GeoSentinel study (38/100,000 travelers) although the actual frequency was probably somewhat underestimated given the very restrictive case definition. Because of the high prevalence of toxocariasis in tropical countries and the inherent risks related to visiting regions with substandard hygiene (exposure to locally prepared food, incidental contacts with animals [23], it is reasonable to include toxocariasis in the differential diagnosis of most travel-related illnesses. However, surprisingly, the rate of *Toxocara* seropositivity in the (suspected) travelers for whom the test was requested (5.5%) was quite similar to that found in suspected autochthonous cases in Denmark (5.5%) [13] or the Netherlands (5–10%) [12] at similar ages. This observation does not support the idea that exotic travel by itself represents a major risk factor for *Toxocara* seropositivity, but since the frequency of clinical toxocariasis was not reported in those studies, comparisons with our findings remain inconclusive. Finally, only one study conducted in the Netherlands has investigated the incidence rate of *Toxocara* infection in travelers by comparing pre- and post-travel serology and found 1.1 seroconversion per 1000 person-months [24]. We confirm here that, even if infrequent, toxocariasis does occur in travelers and has to be considered after any type or duration of travel and from any destination.

Several factors contribute to underdiagnosis of toxocariasis, even in settings with higher resources [25]. Observed symptoms were often little specific and mimicked many other parasitic infections occasionally seen in travelers [26,27]. They were sometimes mild and self-limiting, not always triggering a complete etiological workup. Eosinophilia was rather high within the first weeks after symptom onset but tended to normalize quite rapidly. Laboratory investigations often detected evidence of other helminthic infection, with almost no possibility to discriminate between cross reaction, dual infection or remote exposure. Finally, we observed almost always excellent clinical and laboratory responses to albendazole, which is liberally used as empiric anti-helminthic treatment in travel medicine [28]. Clinicians often tend therefore to consider the specific diagnosis of toxocariasis as somehow difficult and of secondary importance.

Morbidity of toxocariasis should however not be underestimated. In the present series, most patients experienced a protracted illness, also because diagnosis was often delayed. Admission was necessary for three patients (10%), including both neurological cases for whom the diagnosis was particularly challenging. The clinical features of transverse myelitis had indeed developed insidiously, with no concomitant systemic symptoms and no blood eosinophilia at diagnosis, but well laboratory findings indicating an eosinophilic meningitis and non-specific alterations at the Magnetic Resonance Imaging of the spine [29–32]. Both cases did not fully recover despite maximal anti-helminthic therapy and prolonged corticosteroids. For all other cases however, administration of a 5-day course of albendazole was, when tolerated, immediately beneficial, suggesting that the current recommended practice is adequate [8]. This was not the case for ivermectin treatment with which clinical failures were observed [33]. Of note, no clinical exacerbation was observed during anti-parasitic treatment in contrast with what often occurs in other helminthic infections with larval invasion such as acute schistosomiasis [26]. Finally, we did not observe any case of ocular toxocariasis, but such cases are often serologically negative (due to low parasite load) and usually diagnosed in specialized ophthalmologic clinics [34].

In conclusion, symptomatic and asymptomatic toxocariasis was sporadically diagnosed in international travelers attending our center and had clinical and laboratory features overlapping those of many other tropical infections. In the present series, morbidity was non negligible and occasionally severe. A standard 5-day course of albendazole provided substantial clinical benefit without evidence of clinical exacerbation. Research is needed to develop antigen-based tests that would better reflect the disease activity both for diagnostic and monitoring purposes in clinical care.

## Supporting Information

S1 Checklist STROBE checklist. (DOCX)
