# Supplementary material for: Toxocariasis Diagnosed in International Travelers at the Institute of Tropical Medicine, Antwerp, Belgium, from 2000 to 2013
Source: PLoS Negl Trop Dis. 2015 Mar 6;9(3):e0003559. doi: 10.1371/journal.pntd.0003559 (PMC4351981; doi:10.1371/journal.pntd.0003559)
Supplement: S1 Checklist — (DOCX) [file pntd.0003559.s001.docx]

|  | Item No | Recommendation |
| --- | --- | --- |
| **Title and abstract** | 1 | (*a*) Indicate the study’s design with a commonly used term in the title or the abstract |
|  |  | *In this study, we reviewed all cases of asymptomatic and symptomatic toxocariasis diagnosed during post-travel consultations at the reference travel clinic of the Institute of Tropical Medicine, Antwerp, Belgium* |
|  |  | (*b*) Provide in the abstract an informative and balanced summary of what was done and what was found 🡪 cfr. abstract |
| Introduction | | |
| Background/rationale | 2 | Explain the scientific background and rationale for the investigation being reported  *Although many susceptible European travelers are assumed to be at increased risk of exposure to Toxocara spp. during a stay in highly endemic developing countries, frequency and presentation of toxocariasis are largely unknown in this population* |
| Objectives | 3 | State specific objectives, including any prespecified hypotheses  *In the present study, we aimed to assess the frequency, presentation and outcome of toxocariasis diagnosed among travelers and migrants presenting at the travel clinic of the Institute of Tropical Medicine of Antwerp, Belgium*. |
| Methods | | |
| Study design | 4 | Present key elements of study design early in the paper  *Retrospective study with extraction of data from laboratory registries and patient files* |
| Setting | 5 | Describe the setting, locations, and relevant dates, including periods of recruitment, exposure, follow-up, and data collection  *A query was first undertaken in the database of the Central Laboratory of Clinical Biologyof the Institute of Tropical Medicine, Antwerp (ITMA), to retrieve all results of Toxocara serology requested in patients having attended the travel clinic of the ITMA from 2000 to 2013* |
| Participants | 6 | (*a*) Give the eligibility criteria, and the sources and methods of selection of participants. Describe methods of follow-up  *The medical records of all travelers and migrants found with a positive Toxocara serology during this period were then reviewed. Relevant clinical and laboratory data were extracted, de-identified and entered in a Microsoft Access 2010 database. Patients were included according strict case definitions described in details in the text.* |
|  |  | (*b*) For matched studies, give matching criteria and number of exposed and unexposed  *NA* |
| Variables | 7 | Clearly define all outcomes, exposures, predictors, potential confounders, and effect modifiers. Give diagnostic criteria, if applicable  *Variables included: demographic data, month and year of first Toxocara positive serology, most recent travel destination, time of symptom onset after travel, duration of symptoms before consultation, clinical presentation, result of the chest X-rays if requested, absolute blood eosinophil count (and percentage of white blood cell count), Toxocara antibody optical density result, results of parasitological and serological tests prescribed by the physician targeting other helminths according to epidemiological relevance (Ascaris spp., Echinococcus granulosus, Fasciola spp., Filaria spp., Schistosoma spp., Taenia solium, Strongyloides stercoralis, Trichinella spp. and Anisakis simplex), administered treatment(s), clinical and laboratory evolution and outcome.*  *Diagnosis of Toxocara-associated VLM was considered as highly probable when the following criteria were all fulfilled: (1) positive Toxocara serology (entry criteria) AND (2) presence of any systemic symptom compatible with toxocariasis (including fever, respiratory signs such as wheezing, dry cough, dyspnea or an infiltrate on the chest X-ray, abdominal symptoms as abdominal pain, vomiting, diarrhea or hepatomegaly, neurological signs such as focal deficit or encephalopathy, and ocular signs such visual disturbances), with or without dermatological symptoms such as pruritus, urticarial rash or angio-edema AND (3) blood eosinophilia (defined as an absolute blood eosinophil count above 500/µL,or > 7% of the white blood cell count at first presentation [17,18]), or presence of eosinophils in another relevant fluid/tissue) AND (4) reasonable exclusion of alternative diagnosis. Diagnosis of VLM was considered definitive when all 4 criteria were present together with unequivocal Toxocara seroconversion documented on paired serum samples. Diagnosis of asymptomatic toxocariasis relied on the presence of blood eosinophilia at presentation in asymptomatic Toxocara-seropositive individuals and no evidence of other infection likely to explain the eosinophilia.*  *In accordance with the case definitions, we finally excluded from this study all patients with parasitological or serological evidence of another infection or clinically suspect of alternative diagnosis (such as allergy, scabies,…).* |
| Data sources/ measurement | 8* | For each variable of interest, give sources of data and details of methods of assessment (measurement). Describe comparability of assessment methods if there is more than one group  *From 2000 to 2009, the serological diagnosis of toxocariasis was performed with a commercial anti-TES IgG enzyme-linked immunosorbent assay (ELISA) (Toxocaracanis, Bordier Affinity Products SA, Crissier, Switzerland) according to theinstructions of the manufacturer. The assay’s threshold for positivity was set as “weak positive” (from 2000 to 2009) and at 1.0 (measured by optical density, since 2010 onwards) for use in clinical settings.* |
| Bias | 9 | Describe any efforts to address potential sources of bias  *We have divided the patients with positive Toxocara serology in 4 groups of patients according to symptoms and eosinophilia. And we then apply a very restrictive case definition of toxocariasis to be sure to include almost exclusively true cases for the clinical description.* |
| Study size | 10 | Explain how the study size was arrived at  *All results of Toxocara serology requested in patients having attended the travel clinic of the ITMA from 2000 to 2013 (convenient study sample)* |
| Quantitative variables | 11 | Explain how quantitative variables were handled in the analyses. If applicable, describe which groupings were chosen and why  *We categorized all reviewed Toxocara-seropositive cases in four groups according to the clinical reasons for requesting Toxocara serology: 1) presence of symptoms compatible with VLM combined with eosinophilia; 2) asymptomatic eosinophilia; 3) symptoms compatible with VLM without eosinophilia; and 4) possible exposure/no clear reason (Fig 1). We included only the groups 1 and 2for further analysis, to* comply with our case definitions |
| Statistical methods | 12 | (*a*) Describe all statistical methods, including those used to control for confounding  *Differences were compared using Student’s t-test or Mann-WhitneyU-test, when appropriate, for continuous outcome and chi-square and Fisher’s exact tests for categorical outcomes.* |
|  |  | (*b*) Describe any methods used to examine subgroups and interactions  *See item 12 (a) above* |
|  |  | (*c*) Explain how missing data were addressed  *Of 190 travelers with positive Toxocara serology, 3 medical records were incomplete (cfr. Fig.1). These files were excluded from the analysis* |
|  |  | (*d*) If applicable, explain how loss to follow-up was addressed  *In Table 1, we give comments on the evolution and mention loss to follow-up of patients. Loss to follow-up is also addressed in the Results section.* |
|  |  | (*e*) Describe any sensitivity analyses  *NA* |
| Results | | |
| Participants | 13* | (a) Report numbers of individuals at each stage of study—eg numbers potentially eligible, examined for eligibility, confirmed eligible, included in the study, completing follow-up, and analysed  *From January 2000 to August 2013, 3436 Toxocara serological tests were ordered for diagnostic purpose in post-travel care by the ITMA physicians. Of these tests, 190 (5.5%) had positiveanti-TES IgG (Fig 1). Of 187 patients with complete clinical data, 44 had VLM symptoms and eosinophilia (group 1), 35 were found with asymptomatic eosinophilia (group 2) and 54 had symptoms compatible with VLM but no eosinophilia (group 3, excluded from the study); for the remaining 54 individuals with no symptoms and no eosinophilia, the reason for Toxocara testing was considered as unclear (group 4, also excluded from further analysis).*  *In the groups 1 and 2, after exclusion of the cases with alternative diagnosis or possible co-infection, a diagnosis of human toxocariasis was retained in 28 patients, including Toxocara-associated VLM in 23 and asymptomatic toxocariasis in 5 (Fig 1). Diagnosis of VLM was considered as definitive in 7 cases for whom seroconversion was observed. Histological examination was not performed in any case. Of note alternative diagnoses in the excluded cases of groups 1 and 2 (n=51) were mainly strongyloidiasis(n=15), allergic reaction (n=7), filarial infection (n=7), Ancylostoma/Ascaris infection (n=7) and schistosomiasis (n=6). Suspicion of co-infection was also frequent.* |
|  |  | (b) Give reasons for non-participation at each stage *See Fig.1* |
|  |  | (c) Consider use of a flow diagram *See Fig.1* |
| Descriptive data | 14* | (a) Give characteristics of study participants (eg demographic, clinical, social) and information on exposures and potential confounders  *See Table 1, including comments*  *The clinical presentation of the 28 cases is detailed in Table 1. Cases were evenly distributed throughout the study period with no cluster phenomenon.*  *All patients were adult travelers born or residing in Europe, except one Ethiopian child evaluated after adoption and one Lebanese adult living in the Democratic Republic of the Congo. Mean age was 46 years (range: 4-68 years) with a male/female ratio of 0.87. Regions of most recent travels (and presumed exposure) were North and sub-Saharan Africa in 10 patients, Southern and Southeast Asia in 9 and Southern Europe (including Turkey) in 6; for 3 patients (numbers 18, 19 and 26), the continent of acquisition could not be traced with certainty because of multiple travel destinations within a short timeframe. Duration of travel was less than 1 month in 12 (43%) patients and more than 3 months in 10 (36%).*  *For the 23 cases presenting with VLM, symptoms started during the stay abroad in 11 (48%) or developed within 3 weeks in average (range: 0-8 weeks) after return. For one patient (number 11), the dates of recent travel were not clearly reported. Clinical manifestations included abdominal complaints in 11 (48%) patients, respiratory symptoms and skin abnormalities in 10 (43%) each and fever in 9 (39%); in most cases, symptoms were combined or developed sequentially. Radiological pneumonia was found in 5 patients and one of them (number 22) had to be admitted elsewhere because of the severity of respiratory symptoms. Two patients (numbers 11 and 16) presented with progressive neurological features of transverse myelitisbut no other symptoms. In both cases, the diagnosis was made by the demonstration of an increased eosinophil count and of anti-TES IgG in the cerebrospinal fluid, in the absence of another etiology (Table 1).*  *For the 21 patients without neurological complications, median duration of symptoms before first documented medical evaluation at ITMA or elsewhere was 3 weeks (range 5 days- 4 months), while both patients with neurological toxocariasis were diagnosed several months (2,5 and 7 respectively) after symptom onset.* |
|  |  | (b) Indicate number of participants with missing data for each variable of interest  *See Fig.1(only 3 on 190)* |
|  |  | (c) Summarise follow-up time (eg, average and total amount)  *For the 21 patients without neurological complications, median duration of symptoms before first documented medical evaluation at ITMA or elsewhere was 3 weeks (range 5 days- 4 months), while both patients with neurological toxocariasis were diagnosed several months (2,5 and 7 respectively) after symptom onset.* |
| Outcome data | 15* | Report numbers of outcome events or summary measures over time  *See Table 1* |
| Main results | 16 | (*a*) Give unadjusted estimates and, if applicable, confounder-adjusted estimates and their precision (eg, 95% confidence interval). Make clear which confounders were adjusted for and why they were included  *The study output is mostly a detailed clinical description of a series of 28 patients with toxocariasis (Table 1). Few comparisons were done between subgroups, except for blood eosinophilia (here under)*  *At first presentation, the median blood eosinophil count was 1720/µL (range: 510-14160) in the 21 VLM cases without neurological complication and 2080/µL (range: 1100-2970) in the 5 asymptomatic cases. Of note, the median eosinophil count was significantly lower in the VLM patients presenting more than 4 weeks after symptom onset than in those consulting earlier (700/µL versus 2340/µL, p<0.001).* |
|  |  | (*b*) Report category boundaries when continuous variables were categorized  *NA* |
|  |  | (*c*) If relevant, consider translating estimates of relative risk into absolute risk for a meaningful time period  *NA* |
| Other analyses | 17 | Report other analyses done—eg analyses of subgroups and interactions, and sensitivity analyses  *Not really applicable for this descriptive clinical study. Some information on subgroups is provided but there is no sensitivity analysis.* |
| Discussion | | |
| Key results | 18 | Summarise key results with reference to study objectives  *We describe here a case series of 28 patients diagnosed with toxocariasis acquired from all over the world. Clinical presentation was extremely varied and resembled that of many other endemic helminth infections. Morbidity was important and complications sometimes serious. …*  *In conclusion, symptomatic and asymptomatic toxocariasis was sporadically diagnosed in international travelers attending our center and had clinical and laboratory features overlapping those of many other tropical infections. In the present series, morbidity was non negligible and occasionally severe. A standard 5-day course of albendazole provided substantial clinical benefit without evidence of clinical exacerbation. Research is needed to develop antigen-based tests that would better reflect the disease activity both for diagnostic and monitoring purposes in clinical care.* |
| Limitations | 19 | Discuss limitations of the study, taking into account sources of potential bias or imprecision. Discuss both direction and magnitude of any potential bias  *This study has many limitations. It was indeed a retrospective single-center study conducted in a reference travel clinic, meaning that collection of data was not systematic and that findings may not be generalizable to all clinical settings. For instance, some cases with longer incubation period may have been directly attended in the primary care setting given the fact that the link between symptoms and travel had become less obvious. Also our observations are not transposable as such to the features observed in autochthonous toxocariasis sporadically seen in Belgium or elsewhere, mainly in children.. Other limitations could be related to the restrictive case definition that may have missed several true cases without eosinophilia (as sometimes observed in milder cases of “covert/common” toxocariasis) or with negative Toxocara serology (during the serological window period or because sensitivity does not reach 100%, in particular in low burden infection such as ocular toxocariasis). In the same line, some true toxocariasis cases may have been disregarded just because serological tests against other helminths were also positive, either by cross-reactivity or as part of infection with multiple parasites. Conversely, false positive result was also possible if anti-Toxocara seropositivity was just reflecting remote exposure or cross-reaction while another helminthic infection was missed during the workup. The commercial test we used is widely considered as an adequate screening tool for clinical practice but indeed detects anti-TES IgG that may persist for years; IgM or IgE-based serological tests that could better discriminate recent infection are not routinely available.*  *On the other side, for European travelers with little previous exposure to parasites, cross-reaction is probably not a major issue, since test threshold has been set at value providing good specificity. In addition, the consistent clinical and laboratory diagnostic approach by a stable group of expert physicians throughout the study probably reflected the best accuracy that can be obtained in routine care. Finally, in this series, infection was most likely acquired abroad since symptoms developed during or shortly after travel, but infection in Belgium before travel or after return cannot be fully excluded.* |
| Interpretation | 20 | Give a cautious overall interpretation of results considering objectives, limitations, multiplicity of analyses, results from similar studies, and other relevant evidence  *The observation of toxocariasis in travelers is not surprising, although poorly studied so far. In a 10-year retrospective study in Spain, Toxocara antibodies were detected in 31 (4.9%) of 634 Latin American migrants and VLM was diagnosed in 4 of them, but this was in a particular segment of the travel population. With 28 highly probable/definitive toxocariasis cases diagnosed in about 85,000 travelers during the 13-year study period (33/100,000 travelers), our findings are in line with the recent multicenter GeoSentinel study (38/100,000 travelers) although the actual frequency was probably somewhat underestimated given the very restrictive case definition. Because of the high prevalence of toxocariasis in tropical countries and the inherent risks related to visiting regions with substandard hygiene (exposure to locally prepared food, incidental contacts with animals, it is reasonable to include toxocariasis in the differential diagnosis of most travel-related illnesses. However, surprisingly, the rate of Toxocara seropositivity in the (suspected) travelers for whom the test was requested (5.5%) was quite similar to that found in suspected autochthonous cases in Denmark (5.5%)or the Netherlands (5-10%) at similar ages. This observation does not support the idea that exotic travel by itself represents a major risk factor for Toxocara seropositivity, but since the frequency of clinical toxocariasis was not reported in those studies, comparisons with our findings remain inconclusive. Finally, only one study conducted in the Netherlands has investigated the incidence rate of Toxocara infection in travelers by comparing pre- and post-travel serology and found 1.1 seroconversion per 1000 person-months. We confirm here that, even if infrequent, toxocariasis does occur in travelers and has to be considered after any type or duration of travel and from any destination.*  *Several factors contribute to underdiagnosis of toxocariasis, even in settings with higher resources[25]. Observed symptoms were often little specific and mimicked many other parasitic infections occasionally seen in travelers. They were sometimes mild and self-limiting, not always triggering a complete etiological workup. Eosinophilia was rather high within the first weeks after symptom onset but tended to normalize quite rapidly. Laboratory investigations often detected evidence of other helminthic infection, with almost no possibility to discriminate between cross reaction, dual infection or remote exposure. Finally, we observed almost always excellent clinical and laboratory responses to albendazole, which is liberally used as empiric anti-helminthic treatment in travel medicine. Clinicians often tend therefore to consider the specific diagnosis of toxocariasis as somehow difficult and of secondary importance.* |
| Generalisability | 21 | Discuss the generalisability (external validity) of the study results  *It was indeed a retrospective single-center study conducted in a reference travel clinic, meaning that collection of data was not systematic and that findings may not be generalizable to all clinical settings. For instance, some cases with longer incubation period may have been directly attended in the primary care setting given the fact that the link between symptoms and travel had become less obvious. Also our observations are not transposable as such to the features observed in autochthonous toxocariasis sporadically seen in Belgium or elsewhere, mainly in children.* |
| Other information | | |
| Funding | 22 | Give the source of funding and the role of the funders for the present study and, if applicable, for the original study on which the present article is based  *Financial support: none (within the mandate of the Institute)* |

*Give information separately for exposed and unexposed groups.

**Note:** An Explanation and Elaboration article discusses each checklist item and gives methodological background and published examples of transparent reporting. The STROBE checklist is best used in conjunction with this article (freely available on the Web sites of PLoS Medicine at http://www.plosmedicine.org/, Annals of Internal Medicine at http://www.annals.org/, and Epidemiology at http://www.epidem.com/). Information on the STROBE Initiative is available at http://www.strobe-statement.org.
